# Supplementary material for: Artificial Hsp104-mediated systems for re-localizing protein aggregates
Source: Nat Commun. 2023 May 9;14:2663. doi: 10.1038/s41467-023-37706-3 (PMC10169802; doi:10.1038/s41467-023-37706-3)
Supplement: Supplementary file 1 — Supplementary Information [file 41467_2023_37706_MOESM1_ESM.pdf]

Supplementary information

Artificial Hsp104-mediated systems for re-localizing protein aggregates

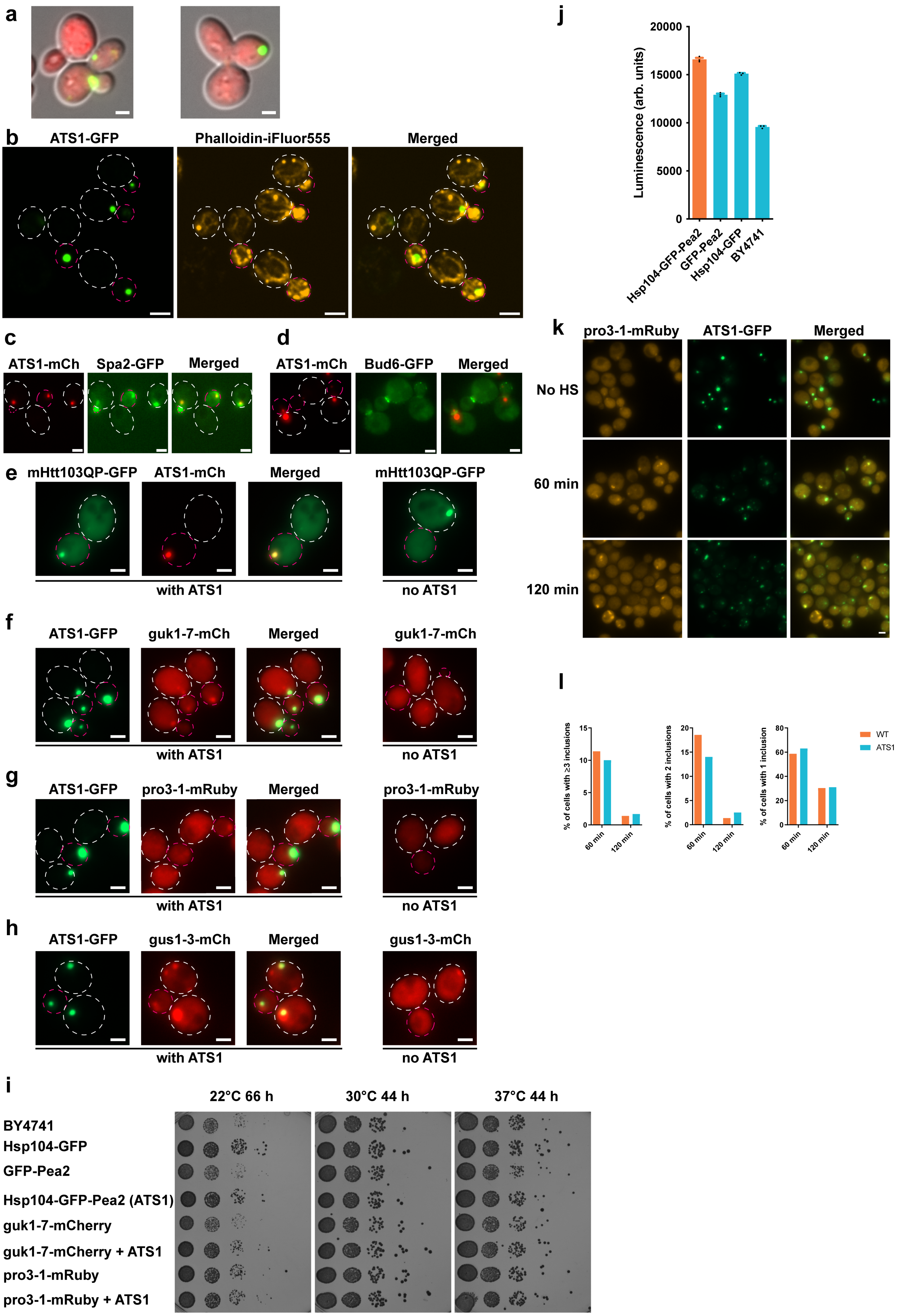

a
b
ATS1-GFP
Phalloidin-iFluor555
Merged
c
ATS1-mCh
Spa2-GFP
Merged
d
ATS1-mCh
Bud6-GFP
Merged
e
mHtt103QP-GFP
ATS1-mCh
Merged
mHtt103QP-GFP
with ATS1
no ATS1
f
ATS1-GFP
guk1-7-mCh
Merged
guk1-7-mCh
with ATS1
no ATS1
g
ATS1-GFP
pro3-1-mRuby
Merged
pro3-1-mRuby
with ATS1
no ATS1
h
ATS1-GFP
gus1-3-mCh
Merged
gus1-3-mCh
with ATS1
no ATS1
i
22°C 66 h
30°C 44 h
37°C 44 h
BY4741
Hsp104-GFP
GFP-Pea2
Hsp104-GFP-Pea2 (ATS1)
guk1-7-mCherry
guk1-7-mCherry + ATS1
pro3-1-mRuby
pro3-1-mRuby + ATS1
j
Luminescence (arb. units)
20000
15000
10000
5000
0
Hsp104-GFP-Pea2
GFP-Pea2
Hsp104-GFP
BY4741
k
pro3-1-mRuby
ATS1-GFP
Merged
No HS
60 min
120 min
l
% of cells with ≥3 inclusions
% of cells with 2 inclusions
% of cells with 1 inclusion
60 min
120 min
WT
ATS1

Supplementary Fig. 1 | Generation of an aggregate targeting system to the bud. a Two representative fluorescence microscopy images showing cell division defects using Hsp104-GFP-Myo2 (green). This fusion protein was not used in later experiments. **b** Representative confocal microscopy images of the co-localization of ATS1 (green, GFP-labeled) with actin filaments and patches (orange; stained with phalloidin-iFluor555). **c** Representative fluorescence microscopy images of the partial co-localization of ATS1 (red; mCherry-labeled) with the polarisome protein Spa2 (green, GFP-labeled). **d** Low co-localization of ATS1 (red; mCherry-labeled) with the polarisome protein Bud6 (green, GFP-labeled). **e** Left panel: Representative fluorescence microscopy images of mHtt103QP-GFP aggregate transport into buds with ATS1. mHtt103QP-GFP was expressed constitutively (GPD promoter). Right panel: mHtt103QP aggregation in the absence of ATS1. **f** Representative fluorescence microscopy images of guk1-7-mCherry (temperature-sensitive allele of *GUK1*) aggregate transport into buds with ATS1. The right panel is showing guk1-7-mCherry expression in the absence of ATS1. No heat shock was applied. **g** Representative fluorescence microscopy images of pro3-1-mRuby (temperature-sensitive allele of *PRO3*) aggregate transport into buds with ATS1. The right panel is showing pro3-1-mRuby expression in the absence of ATS1. No heat shock was applied. **h** Representative fluorescence microscopy images of gus1-3-mCherry (temperature-sensitive allele of *GUS1*) aggregate transport into buds with ATS1. The right panel is showing gus1-3-mCherry expression in the absence of ATS1. No heat shock was applied. **i** Serial growth assay of the indicated yeast strains at 22, 30 or 37 °C. **j** Bioluminescent determination of Hsf1 activity. Data are presented as mean values +/- SEM. n = 3 technical replicates. **k** Representative fluorescence microscopy images of pro3-1-mRuby disaggregation at 38 °C in the presence of ATS1-GFP. **l** Quantification of k. n = 1 experiment. All scale bars within this figure represent 2 µm.

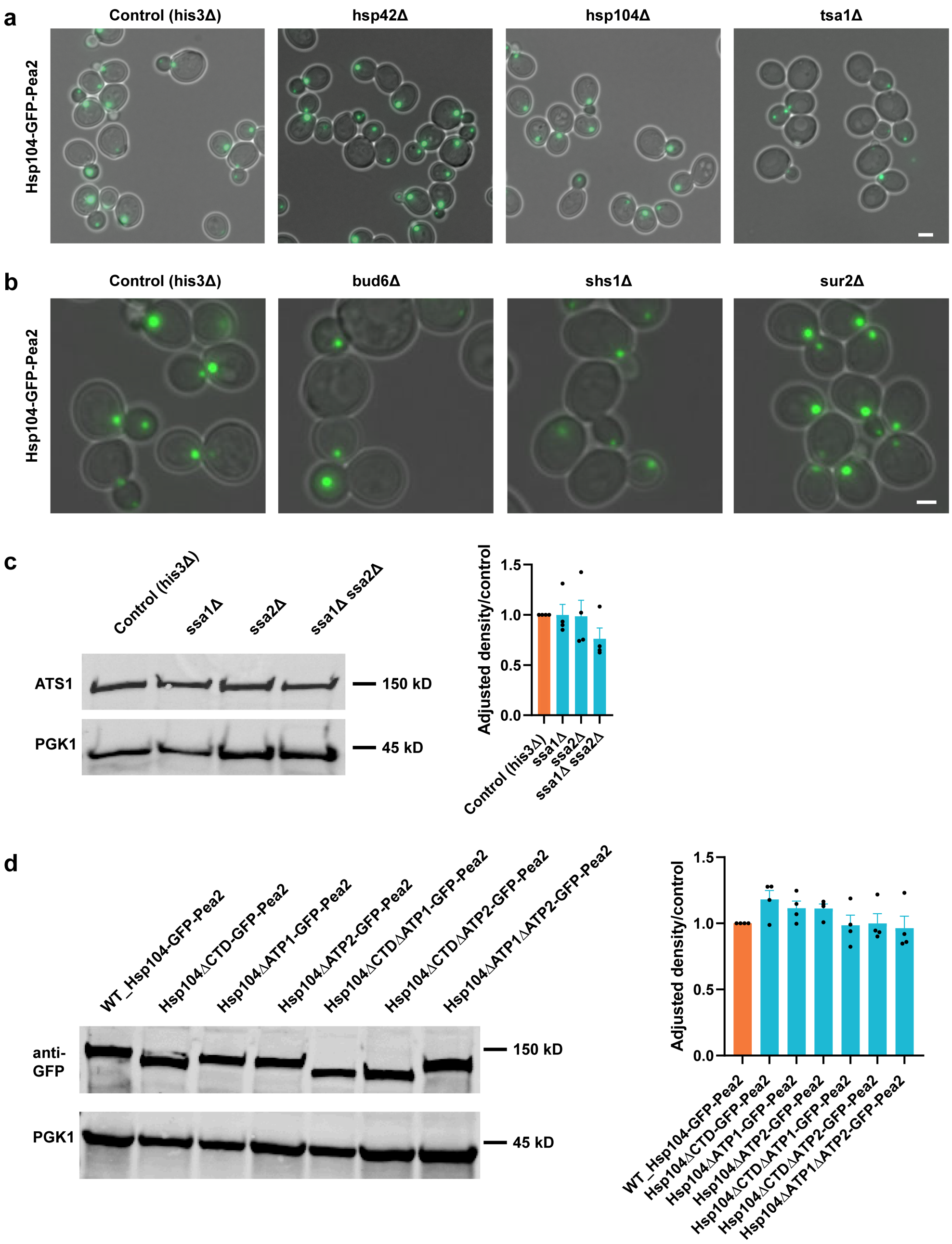

Supplementary Fig. 2 | Genes and genetic modifications affecting ATS1. a ATS1 in deletion mutants of proteostasis genes. **b** ATS1 in deletion mutants of diffusion barrier genes. **c** Left panel: Analysis of expression levels of ATS1 in cells with *SSA1* and/or *SSA2* knockout by Western blot. PGK1 served as loading control. Right panel: Quantification of the band intensity, adjusted to the PGK1 loading control. Data are presented as mean values +/- SEM. n = 4 independent experiments. **d** Left panel: Analysis of expression levels of Hsp104-GFP-Pea2 variants by Western blot. PGK1 served as loading control. Right panel: Quantification of the band intensity, adjusted to the PGK1 loading control. Data are presented as mean values +/- SEM. n = 4 independent experiments. All scale bars within this figure represent 2 μm.

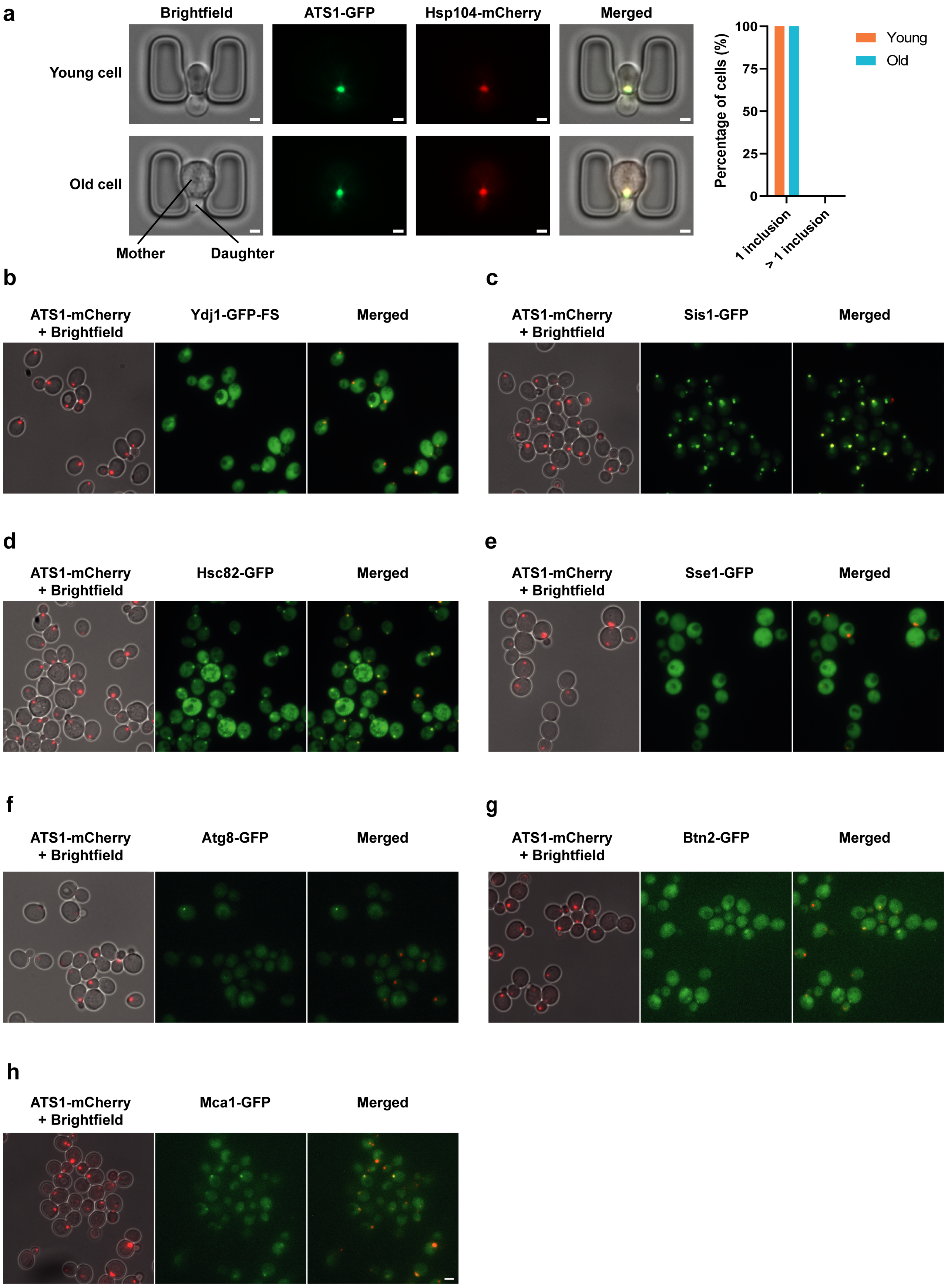
a
Brightfield
ATS1-GFP
Hsp104-mCherry
Merged
Young cell
Old cell
Mother
Daughter
Percentage of cells (%)
100
75
50
25
0
1 inclusion
> 1 inclusion
Young
Old
b
ATS1-mCherry + Brightfield
Ydj1-GFP-FS
Merged
c
ATS1-mCherry + Brightfield
Sis1-GFP
Merged
d
ATS1-mCherry + Brightfield
Hsc82-GFP
Merged
e
ATS1-mCherry + Brightfield
Sse1-GFP
Merged
f
ATS1-mCherry + Brightfield
Atg8-GFP
Merged
g
ATS1-mCherry + Brightfield
Btn2-GFP
Merged
h
ATS1-mCherry + Brightfield
Mca1-GFP
Merged

Supplementary Fig. 3 | Endogenous proteins co-localizing with ATS1. a Representative fluorescence microscopy images of a young and an old cell (15 cell divisions) in a microfluidic chip, expressing Hsp104-GFP-Pea2 (ATS1-GFP). Endogenous Hsp104 was tagged with mCherry. The right panel shows a quantification of the amount of Hsp104-mCherry inclusions per cell (n = 50 cells). **b** Co-localization of ATS1 (red, mCherry-labeled) with endogenous Ydj1 (green, GFP label located between the J domain and the FS domain). **c** Co-localization of ATS1 (red, mCherry-labeled) with endogenous Sis1 (green, GFP-labeled). **d** Co-localization of ATS1 (red, mCherry-labeled) with endogenous Hsc82 (green, GFP-labeled). **e** No co-localization of ATS1 (red, mCherry-labeled) with endogenous Sse1 (green, GFP-labeled). **f** No co-localization of ATS1 (red, mCherry-labeled) with endogenous Atg8 (green, GFP-labeled). **g** Co-localization of ATS1 (red, mCherry-labeled) with endogenous Btn2 (green, GFP-labeled) only after a heat shock (110 min 38°C). **h** Co-localization of ATS1 (red, mCherry-labeled) with endogenous Mca1 (green, GFP-labeled) only after a heat shock (110 min 38°C). All scale bars within this figure represent 2 µm.

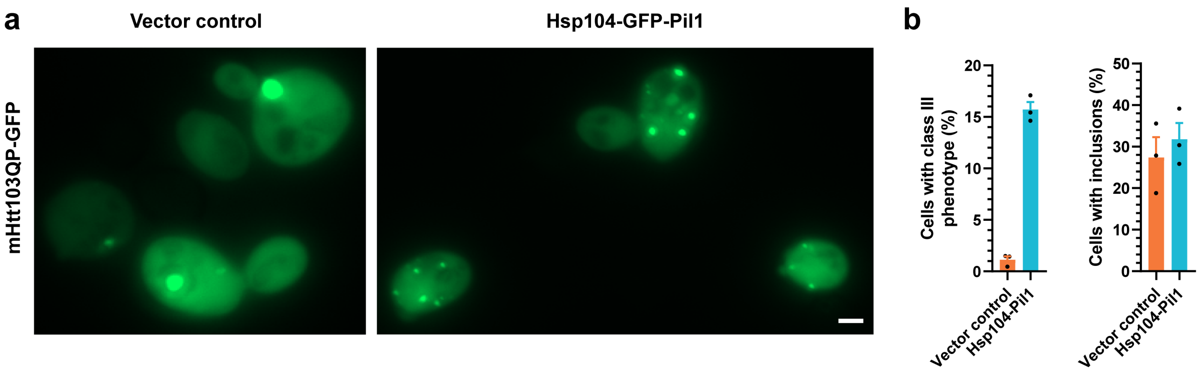

Supplementary Fig. 4 | Manipulating aggregate inclusion site location to other sub-cellular locations. a Representative fluorescence microscopy images showing artificial targeting of Htt103QP-GFP aggregates to eisosomes by using Hsp104-GFP-Pil1. The expression of mHtt103QP-GFP was under the control of the constitutive GPD promoter. **b** Quantification of the amount of mHtt103QP-GFP aggregate inclusions per cell. Data are presented as mean values +/- SEM. n = 3 independent experiments. Class III phenotype: 3 or more aggregate inclusions per cell. All scale bars within this figure represent 2 µm.

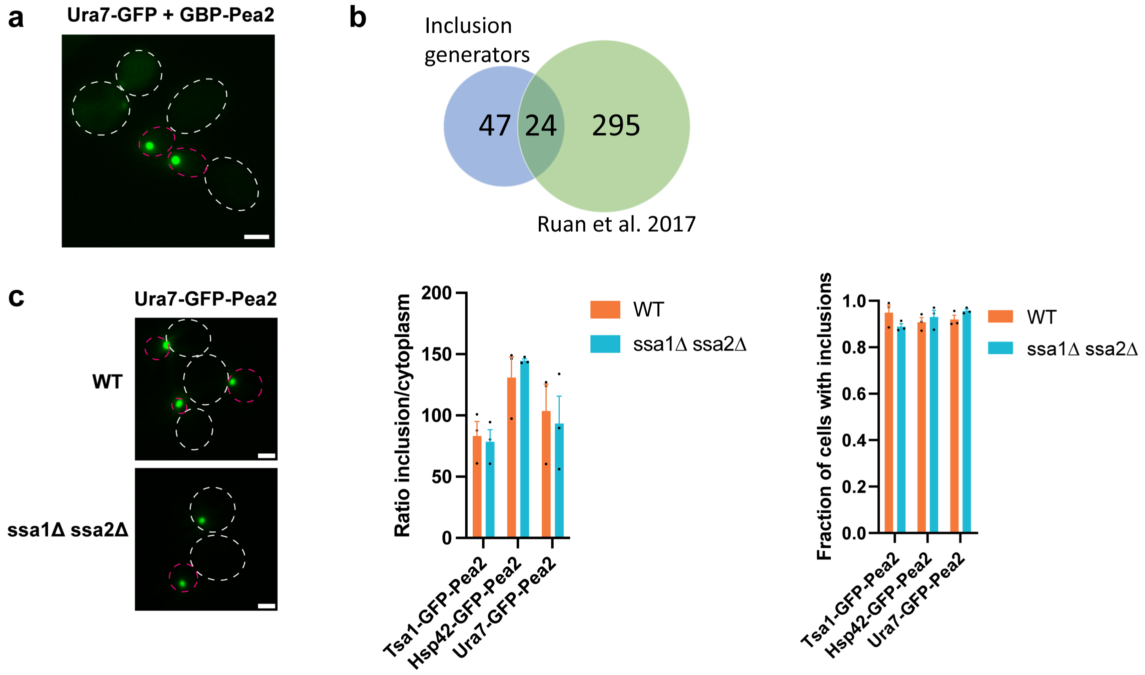

Supplementary Fig. 5 | Generation of different types of artificial inclusions by generating Pea2 chimeras. a Representative fluorescence microscopy image of Ura7-GFP from inclusion generator screen (i.e., in the presence of GBP-Pea2). **b** Venn diagram of the inclusion generator screen hits and proteins found in a screen for heat-shock-induced aggregates from Ruan et al.[1]. **c** Left panel: Representative fluorescence microscopy images showing the effect of double deletion of the Hsp70 genes *SSA1* and *SSA2* on formation of artificial Ura7-GFP-Pea2 inclusions. Cells were grown at 22 °C. Middle panel: Quantifications of the GFP intensity ratio of inclusion to cytoplasm. Right panel: Quantifications of the fraction of cells with ATS1 inclusions. Data are presented as mean values +/- SEM. n = 3 independent experiments. All scale bars within this figure represent 2 µm.

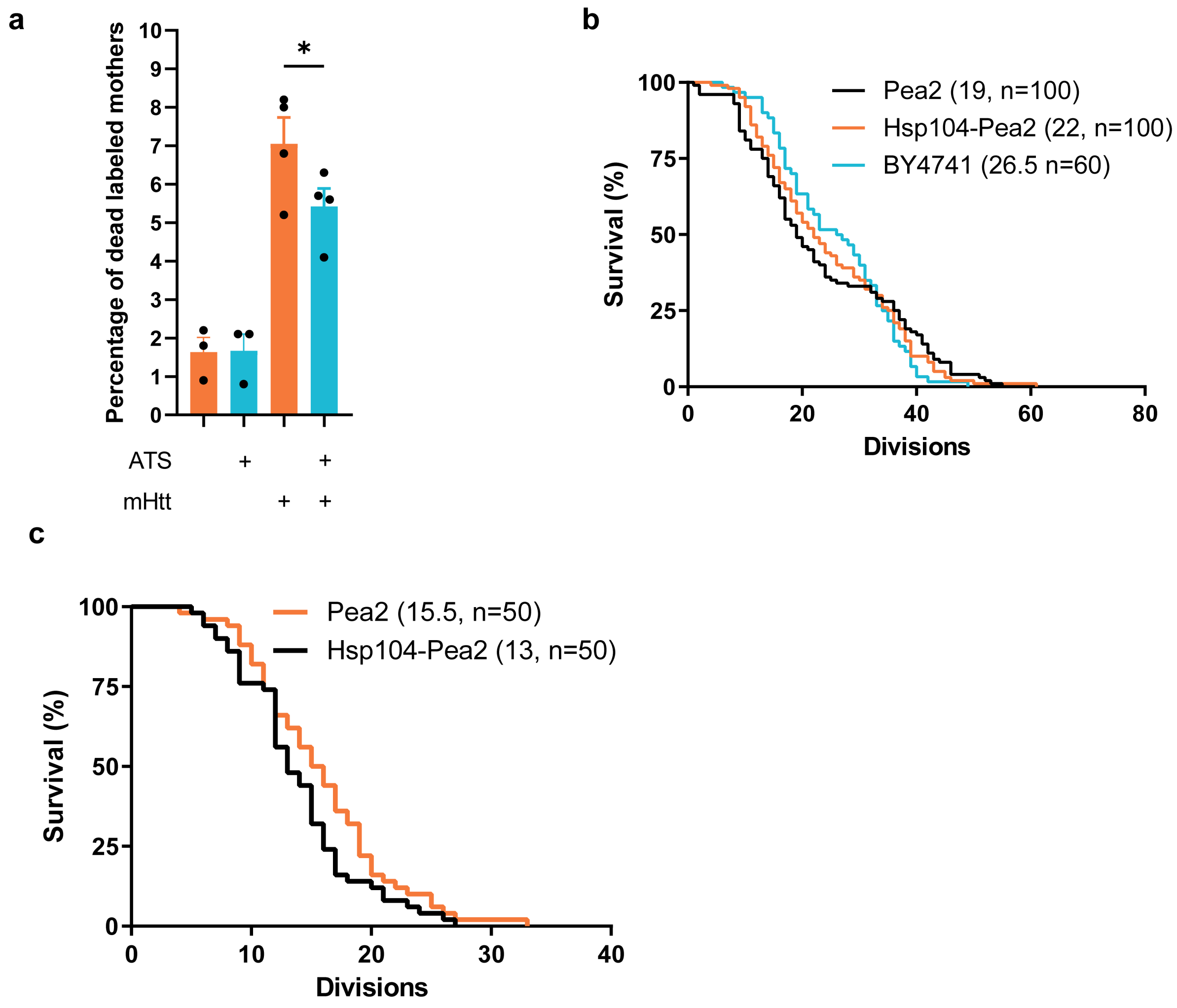

Supplementary Fig. 6 | Cellular effects of exporting protein aggregates from mother cells. a Cell death analysis by flow cytometry of Cyanine5-labeled mother cells after mHtt103Q expression, depicting total cell death levels. Data are presented as mean values +/- SEM. Without mHtt: n = 3; with mHtt: n = 4 independent experiments. *t*-test (two-tailed): **p* = 0.0195. **b** Replicative lifespan analysis via microdissection of cells constitutively expressing ATS1 (*ADH1* promoter). Median lifespan is indicated in parentheses. n = 60-100 cells. **c** Replicative lifespan analysis of cells constitutively expressing ATS1, determined by microfluidics methodology. Median lifespan is indicated in parentheses. n = 50 cells.

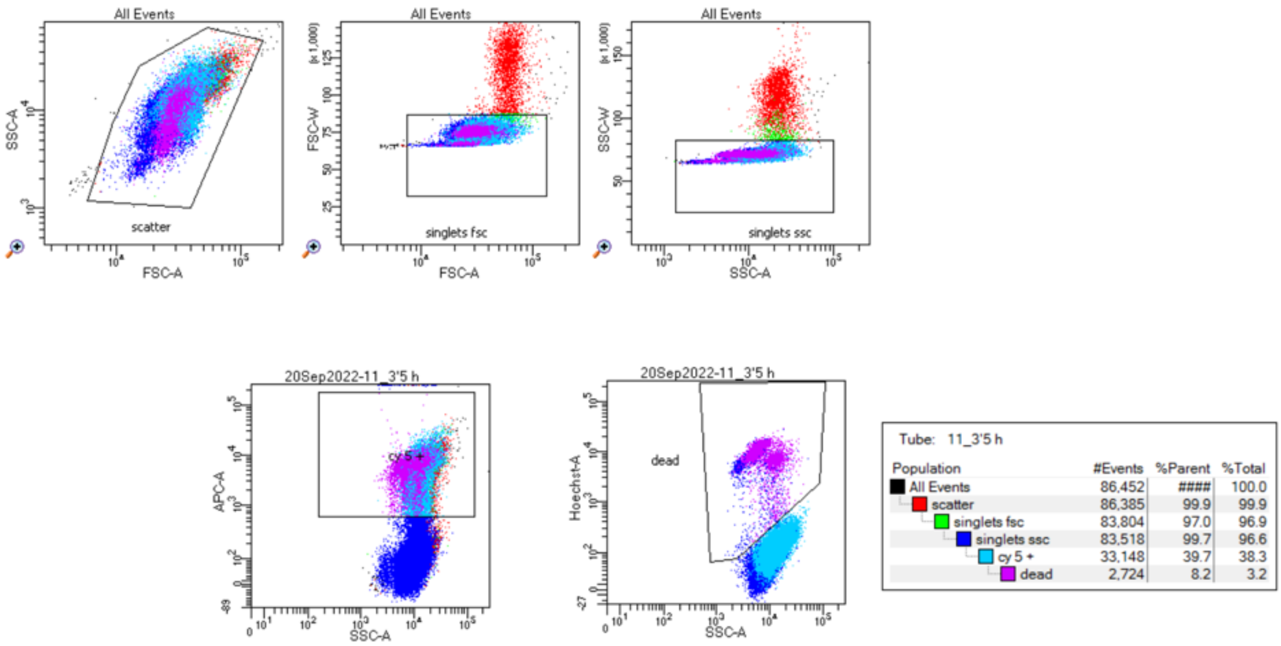

Tube: 11_3'5 h

Population	#Events	%Parent	%Total
All Events	86,452	####	100.0
scatter	86,385	99.9	99.9
singlets fsc	83,804	97.0	96.9
singlets ssc	83,518	99.7	96.6
cy 5 +	33,148	39.7	38.3
dead	2,724	8.2	3.2

Supplementary Fig. 7 | Flow cytometry gating strategies. Cell death analysis by flow cytometry of Cyanine5-labeled mother cells after mHtt103Q expression. SSC-A: side scatter area, FSC-W: forward scatter width, SSC-W: side scatter width, APC-A: Cyanine-5 (Allophycocyanin) channel.

a

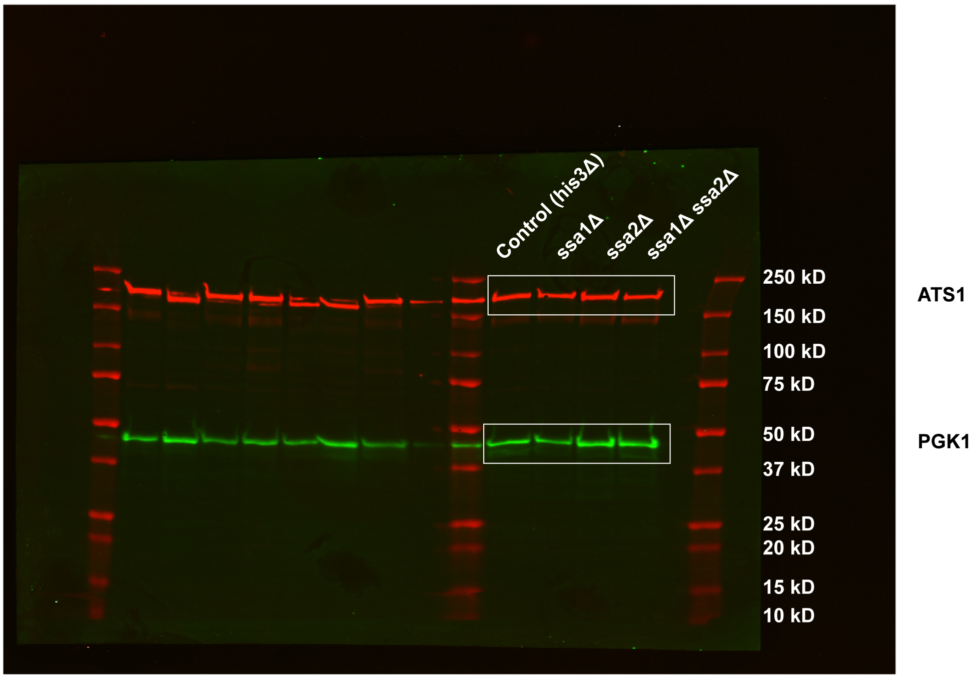

b

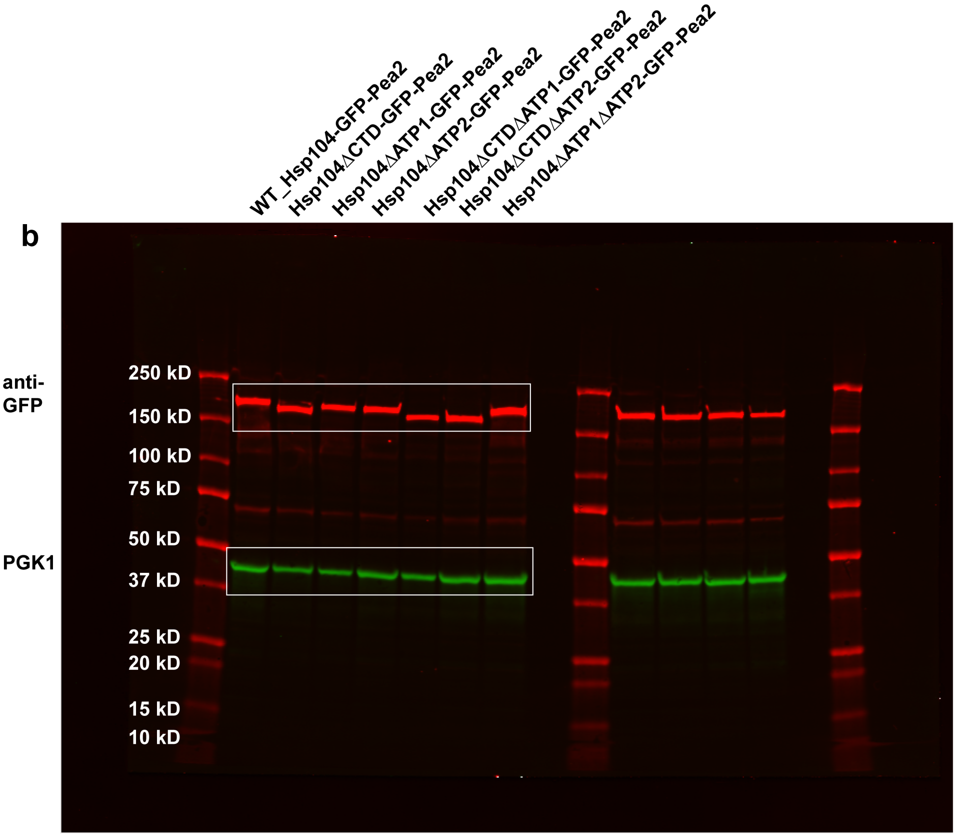

Supplementary Fig. 8 | Uncropped and unprocessed scans of Western blots using an infrared scanner. a Blot from Supplementary Fig. 2c. Red: anti-GFP antibody, green: anti-PGK1 antibody. **b** Blot from Supplementary Fig. 2d. Red: anti-GFP antibody, green: anti-PGK1 antibody.

Supplementary Table 1. Plasmid list.

Plasmid	Replicon	Promoter	Gene	Backbone	Parent	Selection marker	Reference
p416 GPD	CEN/ARS	GPD	GFP	pRS416	p416 GPD	URA3	Krobitsch and Lindquist[2]
p416 25Q GPD	CEN/ARS	GPD	mHtt25QP-GFP	pRS416	p416 GPD	URA3	Krobitsch and Lindquist[2]
p416 103Q GPD	CEN/ARS	GPD	mHtt103QP-GFP	pRS416	p416 GPD	URA3	Krobitsch and Lindquist[2]
pAM09	CEN/ARS	-	-			URA3	Masser, et al.[3]
pAM10	CEN/ARS	CYC1–HSE	NanoLuc			URA3	Masser, et al.[3]
pAF039	-	-	MET15 homology regions	pRS303	pPW411	G418	This study
pAF044	-	Tet3	Hsp104-GFP-Myo2(AA1-1086)	pRS303	pAF039	G418	This study
pAF063	-	ADH1	Hsp104-GFP-Pea2	pRS303	pAF039	G418	This study
pAF066	-	ADH1	GFP-Pea2	pRS303	pAF039	G418	This study
pAF067	-	ADH1	Hsp104-GFP	pRS303	pAF039	G418	This study
pAF070	-	ADH1	Hsp104-GFP-Pea2	pRS303	pPW411	NAT	This study
pAF071	-	ADH1	Hsp104-GFP-Snf7	pRS303	pAF039	G418	This study
pAF075	-	ADH1	Hsp104-mCherry-Pea2	pRS303	pAF039	G418	This study
pAF083	-	ADH1	Hsp104(Δ22)-GFP-Pea2	pRS303	pAF063	G418	This study
pAF084	-	ADH1	Hsp104(Δ38)-GFP-Pea2	pRS303	pAF063	G418	This study
pAF095	-	GPD	Hsp104-GFP-Pil1	pRS303	pAF039	G418	This study
pAF099	-	ADH1	Hsp104(Δ38, ΔATP1)-GFP-Pea2	pRS303	pAF084	G418	This study
pAF100	-	ADH1	Hsp104(Δ38, ΔATP2)-GFP-Pea2	pRS303	pAF084	G418	This study
pAF103	-	ADH1	Hsp104(ΔATP1)-GFP-Pea2	pRS303	pAF063	G418	This study
pAF104	-	ADH1	Hsp104(ΔATP2)-GFP-Pea2	pRS303	pAF063	G418	This study
pAF107	-	ADH1	Hsp104-GFP-p53tet	pRS303	pAF039	G418	This study
pAF111	-	GPD	Hsp104-Pil1	pRS303	pAF095	G418	This study

pAF112	-	ADH1	Hsp104(ΔATP1, ΔATP2)-GFP-Pea2	pRS303	pAF104	G418	This study
pAF118	-	ADH1	GBP-Pea2	pRS303	pAF039	G418	This study
pAF130	-	ADH1	Tsa1(S78D)-GFP-Pea2	pRS303	pAF039	G418	This study
pAF131	-	ADH1	Hsp42(ΔCTD)-GFP-Pea2	pRS303	pAF039	G418	This study
pAF133	-	Tet1	Hsp104-GFP-Pea2	pRS303	pAF039	G418	This study
pAF136	-	ADH1	Tsa1(ΔOI1)-GFP-Pea2	pRS303	pAF039	G418	This study
pAF137	-	ADH1	Tsa1(ΔOI2)-GFP-Pea2	pRS303	pAF039	G418	This study
pAF139	-	ADH1	Hsp42(ΔACD)-GFP-Pea2	pRS303	pAF039	G418	This study
pAF142	-	Tet1	GFP-Pea2	pRS303	pAF039	G418	This study
pAF145	-	ADH1	Hsp42(ΔACD, ΔCTD)-GFP-Pea2	pRS303	pAF039	G418	This study
pAF149	-	ADH1	Hsp42(ΔNTD)-GFP-Pea2	pRS303	pAF039	G418	This study
pAF154	-	ADH1	Hsp42(CTD)-GFP-Pea2	pRS303	pAF039	G418	This study
pAF155	-	ADH1	Hsp42(ACD)-GFP-Pea2	pRS303	pAF039	G418	This study
pAF171	-	Tet1	Hsp104-GFP	pRS303	pAF039	G418	This study
pAF168	SV40	CMV	mCherry-Gag	pcdna4		NeoR/Amp	This study
pAF180	SV40	CMV	Gag	pcdna4		NeoR/Amp	This study
pAF181	SV40	CMV	Hsp104(codon optimized)-mCherry-Gag	pcdna4		NeoR/Amp	This study
pAF195	SV40	CMV	Hsp104(codon optimized)-Gag	pcdna4	pAF181	NeoR/Amp	This study
pGag_eGFP	SV40	CMV	Gag from HIV	pEGFP-N1		NeoR/KanR	Hermida-Matsumoto and Resh[4]
pHttQ119-EYFP	SV40	CMV	mHtt119Q	pEYFP-N1		NeoR/KanR	Rujano, et al.[5]
pPW351	-	GPD	pro3-1-GFP	pRS403		HIS3	Schneider, et al.[6]
pPW390	-	GPD	pro3-1-mRuby2	pRS405		LEU2	Schneider, et al.[6]
pPW411	-	-	MET15 homology regions	pRS303		NAT	This study

pRS413-MYO2	CEN/ARS	MYO2	MYO2	pRS413		HIS3	Catlett and Weisman[7]
pRS416	CEN/ARS	-	-	pRS416		URA3	Sikorski and Hieter[8]
pRS416::GPDp-Htt103QP-mCherry	CEN/ARS	GPD	mHtt103QP-mCherry	pRS416	p416 GPD	URA3	This study
pYES2- GFP	2μ	GAL1	GFP	pYES2		URA3	Prévéral, et al.[9]
pYES2-Htt25Q-GFP	2μ	GAL1	mHtt25Q-GFP	pYES2		URA3	Meriin, et al.[10]
pYES2-Htt25QP-GFP	2μ	GAL1	mHtt25QP-GFP	pYES2		URA3	Meriin, et al.[10]
pYES2-Htt103Q-GFP	2μ	GAL1	mHtt103Q-GFP	pYES2		URA3	Meriin, et al.[10]
pYES2-Htt103QP-GFP	2μ	GAL1	mHtt103QP-GFP	pYES2		URA3	Meriin, et al.[10]
pYES2-Htt25Q-mCherry	2μ	GAL1	mHtt25Q-mCherry	pYES2		URA3	Meriin, et al.[10]
pYES2-Htt25QP-mCherry	2μ	GAL1	mHtt25QP-mCherry	pYES2		URA3	Meriin, et al.[10]
pYES2-Htt103Q-mCherry	2μ	GAL1	mHtt103Q-mCherry	pYES2		URA3	Meriin, et al.[10]
pYES2-Htt103QP-mCherry	2μ	GAL1	mHtt103QP-mCherry	pYES2		URA3	Meriin, et al.[10]

Supplementary Table 2. Yeast strain list.

ID	Name	Genotype	Parent strain	Ingegrated plasmid	Reference
BY4741	BY4741	MATa his3Δ1 leu2Δ0 met15Δ0 ura3Δ0			
guk1-7-mCherry	guk1-7-mCherry	MATa his3Δ1 leu2Δ0 ura3Δ0 met15Δ0 lys2::GPDp-guk1-7-mCherry-PGK1t-LEU2			Schneider, et al.[6]
PW1339	gus1-3-mCherry	MATa his3Δ1 leu2Δ0 ura3Δ0 met15Δ0 lys2::GPDp-gus1-3-mCherry-PGK1t-LEU2			Schneider, et al.[6]
R1158	CMV-tTA	URA3::CMV-tTA MATa his3Δ1 leu2Δ0 met15Δ0			Mnaimneh, et al.[11]
SMH223	ssa1Δ ssa2Δ	MATa ssa1Δ::hphMX ssa2Δ::kanMX4 his3Δ1 LYS2+ leu2Δ0 met15Δ0 ura3Δ0			Hill, et al.[12]
SH164	Tsa1-GFP	MAT a ura3-52 leu2Δ1 trp1Δ63 his3Δ200 lys2ΔBgl hom3-10, ade2Δ1, ade8, hxt13Δ::URA3 TSA1(WT)_GFP::NAT/TRP1 met15Δ0			Hanzen, et al.[13]
SH165	Tsa1_C48S-GFP	MAT a ura3-52 leu2Δ1 trp1Δ63 his3Δ200 lys2ΔBgl hom3-10, ade2Δ1, ade8, hxt13Δ::URA3 tsa1C48S-GFP::NAT/TRP1 cyh2 met15Δ0			Hanzen, et al.[13]
SH166	Tsa1_C171S-GFP	MAT a ura3-52 leu2Δ1 trp1Δ63 his3Δ200 lys2ΔBgl hom3-10, ade2Δ1, ade8, hxt13Δ::URA3 tsaC171S-GFP::NAT/TRP1 cyh2 met15Δ0			Hanzen, et al.[13]
SH169	Tsa1_C48,171S-GFP	MAT a ura3-52 leu2Δ1 trp1Δ63 his3Δ200 lys2ΔBgl hom3-10, ade2Δ1, ade8, hxt13Δ::URA3 tsa1C48S,C171S-GFP::NAT/TRP1 cyh2 met15Δ0			Hanzen, et al.[13]
SH192	Tsa1-GFP	MATα, his3Δ1, leu20, lysΔ0, ura3Δ0 Tsa1-GFP::hph met15Δ0			Hanzen, et al.[13]
SH193	Tsa1_DYF-GFP	MATα, his3Δ1, leu20, lysΔ0, ura3Δ0 Tsa1DYF-GFP::hph met15Δ0			Hanzen, et al.[13]
YAD959	Spa2-GFP	MATa Spa2-GFP-TRP1 his3-Δ200, leu2-3, 112 lys2-801, trp1-Δ63, ura3-52	JD47	pRS304-GFP(S65)T	Dünkler, et al.[14]
YAD2608	Myo2	MATa Spa2-GFP-TRP1 his3-Δ200, leu2-3, 112 lys2-801, trp1-Δ63, MYO2::MYO2-hphNT1	JD47		Dünkler, et al.[14]
YAD2614	Myo2$_{RD}$	MATa Spa2-GFP-TRP1 his3-Δ200, leu2-3, 112 lys2-801, trp1-Δ63, MYO2::myo2$_{R1419D}$-hphNT1	JD47		Dünkler, et al.[14]
yAF023	pro3-1-GFP	MATa Hsp104Δ::kanMX4 his3Δ1::HIS3-pGPD-pro3-1-GFP-tPgk1 leu2Δ0 met15Δ0 ura3Δ0	BY4741	pPW351	This study
yAF100	Hsp104-GFP-Myo2(1-1086)	MATa his3Δ1 leu2Δ0 met15Δ0 ura3Δ0 Gal4::His3-pMyo2-GEV-tPgk1 Ade4::NatMX-rtAct1-pGal1-(rtTA-SE-G72P)-tPgk1 LYS2::Leu2-pGPD-pro3-1-mRuby Met15::TET3p-Hsp104-GFP-Myo2(1-1086)-CYC1t-KanMX	yAF049	pAF044	This study
yAF204	ADH1p-Hsp104-GFP-Pea2	MATa his3Δ1 leu2Δ0 ura3Δ0 met15Δ0::ADH1p-Hsp104-GFP-Pea2-CYC1t-KanMX	BY4741	pAF063	This study

yAF217	Hsp104-mCherry + Hsp104-GFP-Pea2	MATa his3Δ1 leu2Δ0 ura3Δ0 HSP104-mCherry-hphNT1 met15Δ0::ADH1p-Hsp104-GFP-Pea2-CYC1t-KanMX	BY4741	pAF063	This study
yAF219	ADH1p-Hsp104-GFP-Pea2 + Hsp42-mRuby	MATa his3Δ1 leu2Δ0 ura3Δ0 met15Δ0::ADH1p-Hsp104-GFP-Pea2-CYC1t-KanMX HSP42-mRuby-hphNT1	yAF204		This study
yAF221	Hsp104-GFP-Pea2 + pro3-1-mRuby2	MATa his3Δ1 leu2Δ0 ura3Δ0 met15Δ0::ADH1p-Hsp104-GFP-Pea2-CYC1t-KanMX lys2::GPDp-pro3-1-mRuby2-PGK1t-LEU2	yAF204		This study
yAF227	ADH1p-Hsp104-GFP-Pea2 + pYES-GAL1p-Htt103QP-mCherry	MATa his3Δ1 leu2Δ0 ura3Δ0 met15Δ0::ADH1p-Hsp104-GFP-Pea2-CYC1t-KanMX pYES-GAL1p-Htt103QP-mCherry(URA)	yAF204		This study
yAF229	guk1-7-mCherry + ADH1p-Hsp104-GFP-Pea2	MATa his3Δ1 leu2Δ0 ura3Δ0 met15Δ0::ADH1p-Hsp104-GFP-Pea2-CYC1t-KanMX lys2::GPDp-guk1-7-mCherry-PGK1t-LEU2	guk1-7-mCherry		This study
yAF235	GPDp-pro3-1-mRuby2	MATa his3Δ1 leu2Δ0 ura3Δ0 met15Δ0 lys2::GPDp-pro3-1-mRuby2-PGK1t-LEU2	BY4741	pPW390	This study
yAF241	ADH1p-GFP-Pea2	MATa his3Δ1 leu2Δ0 ura3Δ0 met15Δ0::ADH1p-GFP-Pea2-CYC1t-KanMX	BY4741	pAF066	This study
yAF242	ADH1p-Hsp104-GFP	MATa his3Δ1 leu2Δ0 ura3Δ0 met15Δ0::ADH1p-Hsp104-GFP-CYC1t-KanMX	BY4741	pAF067	This study
yAF250	ADH1p-Hsp104-GFP-Pea2 + pAM09	MATa his3Δ1 leu2Δ0 ura3Δ0 met15Δ0::ADH1p-Hsp104-GFP-Pea2-CYC1t-KanMX pAM09(URA)	yAF204		This study
yAF251	ADH1p-Hsp104-GFP-Pea2 + pAM10	MATa his3Δ1 leu2Δ0 ura3Δ0 met15Δ0::ADH1p-Hsp104-GFP-Pea2-CYC1t-KanMX pAM10(URA)	yAF204		This study
yAF252	ADH1p-GFP-Pea2 + pAM09	MATa his3Δ1 leu2Δ0 ura3Δ0 met15Δ0::ADH1p-GFP-Pea2-CYC1t-KanMX pAM09(URA)	yAF241		This study
yAF253	ADH1p-GFP-Pea2 + pAM10	MATa his3Δ1 leu2Δ0 ura3Δ0 met15Δ0::ADH1p-GFP-Pea2-CYC1t-KanMX pAM10(URA)	yAF241		This study
yAF254	ADH1p-Hsp104-GFP + pAM09	MATa his3Δ1 leu2Δ0 ura3Δ0 met15Δ0::ADH1p-Hsp104-GFP-CYC1t-KanMX pAM09(URA)	yAF242		This study
yAF255	ADH1p-Hsp104-GFP + pAM10	MATa his3Δ1 leu2Δ0 ura3Δ0 met15Δ0::ADH1p-Hsp104-GFP-CYC1t-KanMX pAM10(URA)	yAF242		This study
yAF256	BY4741 + pAM09	MATa his3Δ1 leu2Δ0 ura3Δ0 met15Δ0 pAM09(URA)	BY4741		This study
yAF257	BY4741 + pAM10	MATa his3Δ1 leu2Δ0 ura3Δ0 met15Δ0 pAM10(URA)	BY4741		This study
yAF260	ADH1p-Hsp104-GFP-Pea2	MATa his3Δ1 leu2Δ0 ura3Δ0 met15Δ0::ADH1p-Hsp104-GFP-Pea2-CYC1t-NATMX	BY4741		This study
yAF261	bud6Δ + ADH1p-Hsp104-GFP-Pea2	MATa bud6Δ::kanMX4 his3Δ1 leu2Δ0 ura3Δ0 met15Δ0::ADH1p-Hsp104-GFP-Pea2-CYC1t-NATMX	bud6Δ	pAF070	This study
yAF262	shs1Δ + ADH1p-Hsp104-GFP-Pea2	MATa shs16Δ::kanMX4 his3Δ1 leu2Δ0 ura3Δ0 met15Δ0::ADH1p-Hsp104-GFP-Pea2-CYC1t-NATMX	shs1Δ	pAF070	This study
yAF263	sur2Δ + ADH1p-Hsp104-GFP-Pea2	MATa sur2Δ::kanMX4 his3Δ1 leu2Δ0 ura3Δ0 met15Δ0::ADH1p-Hsp104-GFP-Pea2-CYC1t-NATMX	sur2Δ	pAF070	This study

yAF264	hsp42Δ + ADH1p-Hsp104-GFP-Pea2	MATa hsp42Δ::kanMX4 his3Δ1 leu2Δ0 ura3Δ0 met15Δ0::ADH1p-Hsp104-GFP-Pea2-CYC1t-NATMX	hsp42Δ	pAF070	This study
yAF270	ADH1p-Hsp104-GFP-Snf7	MATa his3Δ1 leu2Δ0 ura3Δ0 met15Δ0::ADH1p-Hsp104-GFP-Snf7-CYC1t-KanMX	BY4741	pAF071	This study
yAF277	gus1-3-mCherry + ADH1p-Hsp104-GFP-Pea2	MATa his3Δ1 leu2Δ0 ura3Δ0 met15Δ0::ADH1p-Hsp104-GFP-Pea2-CYC1t-KanMX lys2::GPDp-gus1-3-mCherry-PGK1t-LEU2	PW1339	pAF063	This study
yAF280	ADH1p-Hsp104-GFP-Snf7 + pYES-GAL1p-Htt103QP-mCherry	MATa his3Δ1 leu2Δ0 ura3Δ0 met15Δ0::ADH1p-Hsp104-GFP-Snf7-CYC1t-KanMX pYES-GAL1p-Htt103QP-mCherry(URA)	yAF270		This study
yAF289	Ssa1-GFP + ADH1p-Hsp104-mCherry-Pea2	MATa his3Δ1 leu2Δ0 ura3Δ0 SSA1-GFP-HIS3 met15Δ0::ADH1p-Hsp104-mCherry-Pea2-CYC1t-KanMX	Ssa1-GFP	pAF075	This study
yAF294	pADH1-Hsp104(Δ22)-GFP-Pea2	MATa his3Δ1 leu2Δ0 ura3Δ0 met15Δ0::ADH1p-HSP104(Δ22)-GFP-PEA2-CYC1t-KanMX	BY4741	pAF083	This study
yAF295	pADH1-Hsp104(Δ38)-GFP-Pea2	MATa his3Δ1 leu2Δ0 ura3Δ0 met15Δ0::ADH1p-HSP104(Δ38)-GFP-PEA2-CYC1t-KanMX	BY4741	pAF084	This study
yAF306	GPDp-GFP + ADH1p-Hsp104-mCherry-Pea2	MATa his3Δ1::GPDp-GFP-HIS3 leu2Δ0 ura3Δ0 met15Δ0::ADH1p-Hsp104-mCherry-Pea2-CYC1t-KanMX	GPDp-GFP	pAF075	This study
yAF332	GPDp-Hsp104-GFP-PIL1	MATa his3Δ1 leu2Δ0 ura3Δ0 met15Δ0::GPDp-HSP104-GFP-PIL1-CYC1t-KanMX	BY4741	pAF095	This study
yAF334	ADH1p-Hsp104(Δ38ΔATP1)-eGFP-Pea2	MATa his3Δ1 leu2Δ0 ura3Δ0 met15Δ0::ADH1p-Hsp104(Δ38ΔATP1)-eGFP-PEA2-CYC1t-KanMX	BY4741	pAF099	This study
yAF335	ADH1p-Hsp104(Δ38ΔATP2)-eGFP-Pea2	MATa his3Δ1 leu2Δ0 ura3Δ0 met15Δ0::ADH1p-Hsp104(Δ38ΔATP2)-eGFP-PEA2-CYC1t-KanMX	BY4741	pAF100	This study
yAF346	GPDp-Hsp104-GFP-PIL1 + Htt103QP-mCh.	MATa his3Δ1 leu2Δ0 ura3Δ0 met15Δ0::GPDp-HSP104-GFP-PIL1-CYC1t-KanMX pYES2-GAL1p-Htt103QP-mCherry(URA)	yAF332		This study
yAF350	Ydj1-GFP-FS + ADH1p-Hsp104-mCherry-Pea2	MATa his3Δ1 leu2Δ0 met15Δ0::ADH1p-Hsp104-mCherry-Pea2-CYC1t-KanMX ura3Δ0 YDJ1-GFP-FS-HIS3	Ydj1-GFP-FS	pAF075	This study
yAF351	Sis1-GFP + ADH1p-Hsp104-mCherry-Pea2	MATa his3Δ1 leu2Δ0 met15Δ0::ADH1p-Hsp104-mCherry-Pea2-CYC1t-KanMX ura3Δ0 SIS1-GFP-HIS3	Sis1-GFP	pAF075	This study
yAF352	Btn2-GFP + ADH1p-Hsp104-mCherry-Pea2	MATa his3Δ1 leu2Δ0 met15Δ0::ADH1p-Hsp104-mCherry-Pea2-CYC1t-KanMX ura3Δ0 BTN2-GFP-HIS3	Btn2-GFP	pAF075	This study
yAF353	Mca1-GFP + ADH1p-Hsp104-mCherry-Pea2	MATa his3Δ1 leu2Δ0 met15Δ0::ADH1p-Hsp104-mCherry-Pea2-CYC1t-KanMX ura3Δ0 MCA1-GFP-HIS3	Mca1-GFP	pAF075	This study

yAF355	ssa1Δ ssa2Δ + ADH1p-Hsp104-GFP-Pea2	MATa ssa1Δ::hphMX ssa2Δ::kanMX4 his3Δ1 LYS2+ leu2Δ0 met15Δ0::ADH1p-Hsp104-GFP-Pea2-CYC1t-NATMX ura3Δ0	SMH223	pAF070	This study
yAF357	ADH1p-Hsp104-mCherry-Pea2 + GFP-Atg8	MATa his3Δ1 leu2Δ0 ura3Δ0 met15Δ0::ADH1p-Hsp104-mCherry-Pea2-CYC1t-KanMX GFP-ATG8-HIS3	yAF274	pAF075	This study
yAF358	ADH1p-Hsp104(ΔATP1)-eGFP-Pea2	MATa his3Δ1 leu2Δ0 ura3Δ0 met15Δ0::ADH1p-Hsp104(ΔATP1)-eGFP-PEA2-CYC1t-KanMX	BY4741	pAF103	This study
yAF359	ADH1p-Hsp104(ΔATP2)-eGFP-Pea2	MATa his3Δ1 leu2Δ0 ura3Δ0 met15Δ0::ADH1p-Hsp104(ΔATP2)-eGFP-PEA2-CYC1t-KanMX	BY4741	pAF104	This study
yAF367	ssa1Δ + ADH1p-Hsp104-GFP-Pea2	MATa ssa1Δ::kanMX4 his3Δ1 leu2Δ0 met15Δ0::ADH1p-Hsp104-GFP-Pea2-CYC1t-NATMX ura3Δ0	ssa1Δ	pAF070	This study
yAF368	ssa2Δ + ADH1p-Hsp104-GFP-Pea2	MATa ssa2Δ::kanMX4 his3Δ1 leu2Δ0 met15Δ0::ADH1p-Hsp104-GFP-Pea2-CYC1t-NATMX ura3Δ0	ssa2Δ	pAF070	This study
yAF369	hsp104Δ + ADH1p-Hsp104-GFP-Pea2	MATa hsp104Δ::kanMX4 his3Δ1 leu2Δ0 met15Δ0::ADH1p-Hsp104-GFP-Pea2-CYC1t-NATMX ura3Δ0	hsp104Δ	pAF070	This study
yAF370	npr3Δ + ADH1p-Hsp104-GFP-Pea2	MATa npr3Δ::kanMX4 his3Δ1 leu2Δ0 met15Δ0::ADH1p-Hsp104-GFP-Pea2-CYC1t-NATMX ura3Δ0	npr3Δ	pAF070	This study
yAF371	tco89Δ + ADH1p-Hsp104-GFP-Pea2	MATa tco89Δ::kanMX4 his3Δ1 leu2Δ0 met15Δ0::ADH1p-Hsp104-GFP-Pea2-CYC1t-NATMX ura3Δ0	tco89Δ	pAF070	This study
yAF372	his3Δ + ADH1p-Hsp104-GFP-Pea2	MATa his3Δ::kanMX4 his3Δ1 leu2Δ0 met15Δ0::ADH1p-Hsp104-GFP-Pea2-CYC1t-NATMX ura3Δ0	his3Δ	pAF070	This study
yAF373	Hsc82-GFP + ADH1p-Hsp104-mCherry-Pea2	MATa his3Δ1 leu2Δ0 met15Δ0::ADH1p-Hsp104-mCherry-Pea2-CYC1t-KanMX ura3Δ0 HSC82-GFP-HIS3	Hsc82-GFP	pAF075	This study
yAF374	Tsa1-GFP + ADH1p-Hsp104-mCherry-Pea2	MATa his3Δ1 leu2Δ0 met15Δ0::ADH1p-Hsp104-mCherry-Pea2-CYC1t-KanMX ura3Δ0 SIS1-GFP-HIS3	Tsa1-GFP	pAF075	This study
yAF375	Sse1-GFP + ADH1p-Hsp104-mCherry-Pea2	MATa his3Δ1 leu2Δ0 met15Δ0::ADH1p-Hsp104-mCherry-Pea2-CYC1t-KanMX ura3Δ0 Sse1-GFP-HIS3	Sse1-GFP	pAF075	This study
yAF378	ADH1p-Hsp104-GFP-p53TETD	MATa his3Δ1 leu2Δ0 ura3Δ0 met15Δ0::ADH1p-Hsp104-GFP-p53TETD-CYC1t-KanMX	BY4741	pAF107	This study
yAF385	ADH1p-Hsp104-mCherry-Pea2 + pRS416::GPDp-Htt103QP-GFP	MATa his3Δ1 leu2Δ0 ura3Δ0 met15Δ0::ADH1p-Hsp104-mCherry-Pea2-CYC1t-KanMX pRS416::GPDp-Htt103QP-GFP(URA)	yAF274		This study

yAF386	met15Δ vector control + pRS416	MATa his3Δ1 leu2Δ0 ura3Δ0 met15Δ0::KanMX pRS416(URA)	yAF290		This study
yAF387	met15Δ vector control + pRS416::GPDp-GFP	MATa his3Δ1 leu2Δ0 ura3Δ0 met15Δ0::KanMX pRS416::GPDp-GFP(URA)	yAF290		This study
yAF388	met15Δ vector control + pRS416::GPDp-Htt25Q-GFP	MATa his3Δ1 leu2Δ0 ura3Δ0 met15Δ0::KanMX pRS416::GPDp-Htt25Q-GFP(URA)	yAF290		This study
yAF389	met15Δ vector control + pRS416::GPDp-Htt103QP-GFP	MATa his3Δ1 leu2Δ0 ura3Δ0 met15Δ0::KanMX pRS416::GPDp-GFP(URA)	yAF290		This study
yAF393	GPDp-Hsp104-Pil1 (No GFP)	MATa his3Δ1 leu2Δ0 ura3Δ0 met15Δ0::GPDp-HSP104-PIL1-CYC1t-KanMX	BY4741	pAF111	This study
yAF394	ADH1p-Hsp104ΔATP1,ΔATP2-eGFP-Pea2	MATa his3Δ1 leu2Δ0 ura3Δ0 met15Δ0::ADH1p-Hsp104ΔATP1,ΔATP2-eGFP-PEA2-CYC1t-KanMX	BY4741	pAF112	This study
yAF411	GPDp-Hsp104-Pil1 + pRS416::GPDp	MATa his3Δ1 leu2Δ0 ura3Δ0 met15Δ0::GPDp-HSP104-PIL1-CYC1t-KanMX pRS416::GPDp-GFP(URA)	yAF393		This study
yAF412	GPDp-Hsp104-Pil1 + pRS416::Htt25Q	MATa his3Δ1 leu2Δ0 ura3Δ0 met15Δ0::GPDp-HSP104-PIL1-CYC1t-KanMX pRS416::GPDp-Htt25Q-GFP(URA)	yAF393		This study
yAF413	GPDp-Hsp104-Pil1 + pRS416::Htt103QP	MATa his3Δ1 leu2Δ0 ura3Δ0 met15Δ0::GPDp-HSP104-PIL1-CYC1t-KanMX pRS416::GPDp-Htt103QP-GFP(URA)	yAF393		This study
yAF475	GBP-Pea2 + Htt103QP-mCherry query strain for screen	MATα can1Δ::STE2pr-LEU2 lyp1Δ his3Δ1 leu2Δ0 ura3Δ0 met15Δ::ADH1p-GBP-Pea2-KanMX pRS416::GPDp-Htt103QP-mCherry(URA)	yAF470		This study
yAF477	tsa1Δ + ADH1p-Hsp104-GFP-Pea2	MATa tsa1Δ::KanMX4 his3Δ1 leu2Δ0 met15Δ0::ADH1p-Hsp104-GFP-Pea2-CYC1t-NATMX ura3Δ0	tsa1Δ	pAF070	This study
yAF490	ssa1Δ ssa2Δ + ADH1p-Tsa1-GFP-Pea2	MATa ssa1Δ::hphMX ssa2Δ::kanMX4 his3Δ1 LYS2+ leu2Δ0 met15Δ0::ADH1p-Tsa1-GFP-Pea2-CYC1t-NATMX ura3Δ0	SMH223	pAF128	This study
yAF491	ssa1Δ ssa2Δ + ADH1p-Hsp42-GFP-Pea2	MATa ssa1Δ::hphMX ssa2Δ::kanMX4 his3Δ1 LYS2+ leu2Δ0 met15Δ0::ADH1p-Hsp42-GFP-Pea2-CYC1t-NATMX ura3Δ0		pAF129	
yAF492	Tsa1-GFP + GBP-Pea2	MAT a ura3-52 leu2Δ1 trp1Δ63 his3Δ200 lys2ΔBgl hom3-10, ade2Δ1, ade8, hxt13Δ::URA3 TSA1(WT)_GFP::NAT/TRP1 met15Δ::ADH1p-GFPnanobody-Pea2-KanMX	SH164	pAF118	This study
yAF493	Tsa1_C48S-GFP GBP-Pea2	MAT a ura3-52 leu2Δ1 trp1Δ63 his3Δ200 lys2ΔBgl hom3-10, ade2Δ1, ade8, hxt13Δ::URA3 tsa1C48S-GFP::NAT/TRP1 cyh2 met15Δ::ADH1p-GFPnanobody-Pea2-KanMX	SH165	pAF118	This study
yAF494	Tsa1_C171S-GFP GBP-Pea2	MAT a ura3-52 leu2Δ1 trp1Δ63 his3Δ200 lys2ΔBgl hom3-10, ade2Δ1, ade8, hxt13Δ::URA3 tsaC171S-	SH166	pAF118	This study

		GFP::NAT/TRP1 cyh2 met15Δ::ADH1p-GFPnanobody-Pea2-KanMX			
yAF495	Tsa1_C48,171S-GFP GBP-Pea2	MAT a ura3-52 leu2Δ1 trp1Δ63 his3Δ200 lys2ΔBgl hom3-10, ade2Δ1, ade8, hxt13Δ::URA3 tsa1C48S,C171S-GFP::NAT/TRP1 cyh2 met15Δ::ADH1p-GFPnanobody-Pea2-KanMX	SH169	pAF118	This study
yAF496	Tsa1-GFP + GBP-Pea2	MATα, his3Δ1, leu20, lysΔ0, ura3Δ0 Tsa1-GFP::hph met15Δ::ADH1p-GBP-Pea2-KanMX	SH192	pAF118	This study
yAF497	Tsa1_DYF-GFP GBP-Pea2	MATα, his3Δ1, leu20, lysΔ0, ura3Δ0 Tsa1DYF-GFP::hph met15Δ::ADH1p-GBP-Pea2-KanMX	SH193	pAF118	This study
yAF498	ADH1p-Tsa1-GFP-Pea2-NAT	MATa his3Δ1 leu2Δ0 met15Δ0::ADH1p-Tsa1-GFP-Pea2-CYC1t-NatMX ura3Δ0	BY4741	pAF128	This study
yAF499	ADH1p-Hsp42-GFP-Pea2-NAT	MATa his3Δ1 leu2Δ0 met15Δ0::ADH1p-Hsp42-GFP-Pea2-CYC1t-NatMX ura3Δ0	BY4741	pAF129	This study
yAF500	ADH1p-Tsa1(S78D)-GFP-Pea2	MATa his3Δ1 leu2Δ0 met15Δ0::ADH1p-Tsa1(S78D)-GFP-Pea2-CYC1t-KanMX ura3Δ0	BY4741	pAF130	This study
yAF501	ADH1p-Hsp42(ΔCTD)-GFP-Pea2	MATa his3Δ1 leu2Δ0 met15Δ0::ADH1p-Hsp42(ΔCTD)-GFP-Pea2-CYC1t-KanMX ura3Δ0	BY4741	pAF131	This study
yAF504	ADH1p-Tsa1ΔOI1-GFP-Pea2	MATa his3Δ1 leu2Δ0 met15Δ0::ADH1p-Tsa1_ΔOI1-GFP-Pea2-CYC1t-KanMX ura3Δ0	BY4741	pAF136	This study
yAF505	ADH1p-Tsa1ΔOI2-GFP-Pea2	MATa his3Δ1 leu2Δ0 met15Δ0::ADH1p-Tsa1_ΔOI2-GFP-Pea2-CYC1t-KanMX ura3Δ0	BY4741	pAF137	This study
yAF506	ADH1p-Hsp42(ΔACD)-GFP-Pea2	MATa his3Δ1 leu2Δ0 met15Δ0::ADH1p-Hsp42(ΔACD)-GFP-Pea2-CYC1t-KanMX ura3Δ0	BY4741	pAF139	This study
yAF507	Tet1p-Hsp104GFP-Pea2	URA3::CMV-tTA MATa his3Δ1 leu2Δ0 met15Δ0::TET1p-Hsp104-GFP-Pea2-CYC1t-KanMX	R1158	pAF133	This study
yAF508	Tet1p-GFP-Pea2	URA3::CMV-tTA MATa his3Δ1 leu2Δ0 met15Δ0::TET1p-GFP-Pea2-CYC1t-KanMX	R1158	pAF142	This study
yAF514	ADH1p-Hsp42(ΔACD, ΔCTD)-GFP-Pea2	MATa his3Δ1 leu2Δ0 met15Δ0::ADH1p-Hsp42(ΔACD, ΔCTD)-GFP-Pea2-CYC1t-KanMX ura3Δ0	BY4741	pAF145	This study
yAF517	BY4741 + Hsp42(ΔNTD)	MATa his3Δ1 leu2Δ0 met15Δ0::ADH1p-Hsp42(ΔNTD)-GFP-Pea2-CYC1t-KanMX ura3Δ0	BY4741	pAF149	This study
yAF556	BY4741 + Hsp42_CTD	MATa his3Δ1 leu2Δ0 met15Δ0::ADH1p-Hsp42(CTD)-GFP-Pea2-CYC1t-KanMX ura3Δ0	BY4741	pAF154	This study
yAF557	BY4741 + Hsp42_ACD	MATa his3Δ1 leu2Δ0 met15Δ0::ADH1p-Hsp42(ACD)-GFP-Pea2-CYC1t-KanMX ura3Δ0	BY4741	pAF155	This study
yAF566	Tet1p-Hsp104-GFP	URA3::CMV-tTA MATa his3Δ1 leu2Δ0 met15Δ0::TET1p-Hsp104-GFP-Pea2-CYC1t-KanMX	R1158	pAF171	This study
yAF569	Snf7-GFP + pYES2-Gal1p-Htt103QP-mCh	MATa his3Δ1 leu2Δ0 met15Δ0 ura3Δ0 Snf7-GFP-HIS3 pYES2-GAL1p-Htt103QP-mCherry(URA)	Snf7-GFP		This study
yAF570	Pil1-GFP + pYES2-Gal1p-Htt103QP-mCh	MATa his3Δ1 leu2Δ0 met15Δ0 ura3Δ0 Pil1-GFP-HIS3 pYES2-GAL1p-Htt103QP-mCherry(URA)	Pil1-GFP		This study

yAF600	Bud6-GFP + ADH1p-Hsp104-mCherry-Pea2	MATa his3Δ1 leu2Δ0 met15Δ0::ADH1p-Hsp104-mCherry-Pea2-CYC1t-KanMX ura3Δ0 Bud6-GFP-HIS3	Bud6-GFP	pAF075	This study
yAF601	Myo2-GFP + ADH1p-Hsp104-mCherry-Pea2	MATa his3Δ1 leu2Δ0 met15Δ0::ADH1p-Hsp104-mCherry-Pea2-CYC1t-KanMX ura3Δ0 Myo2-GFP-HIS3	Myo2-GFP	pAF075	This study
yAF602	Myo2-WT + ADH1p-Hsp104-GFP-Pea2	MATa Spa2-GFP-TRP1 his3-Δ200, leu2-3, 112 lys2-801, trp1-Δ63, MYO2::MYO2-hphNT1 met15::ADH1p-Hsp104-GFP-Pea2-CYC1t-KanMX	YAD2608	pAF063	This study
yAF603	Myo2-R1419D + ADH1p-Hsp104-GFP-Pea2	MATa Spa2-GFP-TRP1 his3-Δ200, leu2-3, 112 lys2-801, trp1-Δ63, MYO2::myo2R1419D-hphNT1 met15::ADH1p-Hsp104-GFP-Pea2-CYC1t-KanMX	YAD2614	pAF063	This study
yAF606	ADH1p-Ura7-GFP-Pea2	MATa his3Δ1 leu2Δ0 met15Δ0::ADH1p-Ura7-GFP-Pea2-CYC1t-NATMX ura3Δ0 ura3Δ0 HSP104-mCherry-hphNT1	Hsp104-mCherry	pAF216	This study
yAF614	ssa1Δ ssa2Δ + ADH1p-Ura7-GFP-Pea2	MATa ssa1Δ::hphMX ssa2Δ::kanMX4 his3Δ1 LYS2+ leu2Δ0 met15Δ0::ADH1p-Ura7-GFP-Pea2-CYC1t-NATMX ura3Δ0	SMH223	pAF216	This study
yAF654	Spa2-GFP + ADH1p-Hsp104-mCherry-Pea2	MATa Spa2-GFP-TRP1 his3-Δ200, leu2-3, 112 lys2-801, trp1-Δ63, MYO2::MYO2-hphNT1 met15::ADH1p-Hsp104-mCherry-Pea2-CYC1t-KanMX	YAD959	pAF075	This study
yAJ002	ADH1p-Hsp104-Pea2 + pYES2-Q25-EGFP	MATa his3Δ1 leu2Δ0 ura3Δ0 met15Δ0::ADH1p-Hsp104-Pea2-CYC1t-KanMX pYES2-Q25-EGFP(URA)	yAF275		This study
yAJ003	ADH1p-Hsp104-Pea2 + pYES2-QP25-EGFP	MATa his3Δ1 leu2Δ0 ura3Δ0 met15Δ0::ADH1p-Hsp104-Pea2-CYC1t-KanMX pYES2-Qp25-EGFP(URA)	yAF275		This study
yAJ004	ADH1p-Hsp104-Pea2 + pYES2-Q103-EGFP	MATa his3Δ1 leu2Δ0 ura3Δ0 met15Δ0::ADH1p-Hsp104-Pea2-CYC1t-KanMX pYES2-Q103-EGFP(URA)	yAF275		This study
yAJ005	ADH1p-Hsp104-Pea2 + pYES2-QP103-EGFP	MATa his3Δ1 leu2Δ0 ura3Δ0 met15Δ0::ADH1p-Hsp104-Pea2-CYC1t-KanMX pYES2-Qp103-EGFP(URA)	yAF275		This study
yAJ008	met15Δ vector control + pYES2-EGFP	MATa his3Δ1 leu2Δ0 ura3Δ0 met15Δ0::KanMX pYES2-EGFP(URA)	yAF290		This study
yAJ009	met15Δ vector control + pYES2-Q25-EGFP	MATa his3Δ1 leu2Δ0 ura3Δ0 met15Δ0::KanMX pYES2-Q25-EGFP(URA)	yAF290		This study
yAJ010	met15Δ vector control + pYES2-QP25-EGFP	MATa his3Δ1 leu2Δ0 ura3Δ0 met15Δ0::KanMX pYES2-Qp25-EGFP(URA)	yAF290		This study
yAJ011	met15Δ vector control + pYES2-Q103-EGFP	MATa his3Δ1 leu2Δ0 ura3Δ0 met15Δ0::KanMX pYES2-Q103-EGFP(URA)	yAF290		This study
yAJ012	met15Δ vector control + pYES2-QP103-EGFP	MATa his3Δ1 leu2Δ0 ura3Δ0 met15Δ0::KanMX pYES2-QP103-EGFP(URA)	yAF290		This study

yAJ028	GPDp-Hsp104-Pil1 (No GFP) + pYES2-EGFP	MATa his3Δ1 leu2Δ0 ura3Δ0 met15Δ0::KanMX pRS416::GPDp-Htt103Q-GFP(URA) + pYES2-EGFP	yAF393		This study
yAJ029	GPDp-Hsp104-Pil1 (No GFP) + pYES2-Q25-EGFP	MATa his3Δ1 leu2Δ0 ura3Δ0 met15Δ0::KanMX pRS416::GPDp-Htt103Q-GFP(URA) + pYES2-Q25-EGFP	yAF393		This study
yAJ030	GPDp-Hsp104-Pil1 (No GFP) + pYES2-QP25-EGFP	MATa his3Δ1 leu2Δ0 ura3Δ0 met15Δ0::KanMX pRS416::GPDp-Htt103Q-GFP(URA) + pYES2-QP25-EGFP	yAF393		This study
yAJ031	GPDp-Hsp104-Pil1 (No GFP) + pYES2-Q103-EGFP	MATa his3Δ1 leu2Δ0 ura3Δ0 met15Δ0::KanMX pRS416::GPDp-Htt103Q-GFP(URA) + pYES2-Q103-EGFP	yAF393		This study
yAJ032	GPDp-Hsp104-Pil1 (No GFP) + pYES2-QP103-EGFP	MATa his3Δ1 leu2Δ0 ura3Δ0 met15Δ0::KanMX pRS416::GPDp-Htt103Q-GFP(URA) + pYES2-QP103-EGFP	yAF393		This study
Ydj1-GFP-FS	Ydj1-GFP-FS	MATa his3Δ1 leu2Δ0 met15Δ0 ura3Δ0 YDJ1-GFP-FS-HIS3			Moreno, et al.[15]

Supplementary References

1 Ruan, L. *et al.* Cytosolic proteostasis through importing of misfolded proteins into mitochondria. *Nature* **543**, 443-446 (2017). https://doi.org:10.1038/nature21695

2 Krobitsch, S. & Lindquist, S. Aggregation of huntingtin in yeast varies with the length of the polyglutamine expansion and the expression of chaperone proteins. *Proc Natl Acad Sci U S A* **97**, 1589-1594 (2000). https://doi.org:10.1073/pnas.97.4.1589

3 Masser, A. E., Kandasamy, G., Kaimal, J. M. & Andréasson, C. Luciferase NanoLuc as a reporter for gene expression and protein levels in Saccharomyces cerevisiae. *Yeast* **33**, 191-200 (2016). https://doi.org:https://doi.org/10.1002/yea.3155

4 Hermida-Matsumoto, L. & Resh, M. D. Localization of Human Immunodeficiency Virus Type 1 Gag and Env at the Plasma Membrane by Confocal Imaging. *Journal of Virology* **74**, 8670-8679 (2000). https://doi.org:doi:10.1128/JVI.74.18.8670-8679.2000

5 Rujano, M. A. *et al.* Polarised asymmetric inheritance of accumulated protein damage in higher eukaryotes. *PLoS Biol* **4**, e417 (2006). https://doi.org:10.1371/journal.pbio.0040417

6 Schneider, K. L., Nystrom, T. & Widlund, P. O. Studying Spatial Protein Quality Control, Proteopathies, and Aging Using Different Model Misfolding Proteins in S. cerevisiae. *Front Mol Neurosci* **11**, 249 (2018). https://doi.org:10.3389/fnmol.2018.00249

7 Catlett, N. L. & Weisman, L. S. The terminal tail region of a yeast myosin-V mediates its attachment to vacuole membranes and sites of polarized growth. *Proceedings of the National Academy of Sciences* **95**, 14799 (1998).

8 Sikorski, R. S. & Hieter, P. A system of shuttle vectors and yeast host strains designed for efficient manipulation of DNA in Saccharomyces cerevisiae. *Genetics* **122**, 19-27 (1989). https://doi.org:10.1093/genetics/122.1.19

9 Prévéral, S., Ansoborlo, E., Mari, S., Vavasseur, A. & Forestier, C. Metal(loid)s and radionuclides cytotoxicity in Saccharomyces cerevisiae. Role of YCF1, glutathione and effect of buthionine sulfoximine. *Biochimie* **88**, 1651-1663 (2006). https://doi.org:https://doi.org/10.1016/j.biochi.2006.05.016

10 Meriin, A. B. *et al.* Endocytosis machinery is involved in aggregation of proteins with expanded polyglutamine domains. *The FASEB Journal* **21**, 1915-1925 (2007). https://doi.org:https://doi.org/10.1096/fj.06-6878com

11 Mnaimneh, S. *et al.* Exploration of Essential Gene Functions via Titratable Promoter Alleles. *Cell* **118**, 31-44 (2004). https://doi.org:https://doi.org/10.1016/j.cell.2004.06.013

12 Hill, S. M., Hao, X., Liu, B. & Nystrom, T. Life-span extension by a metacaspase in the yeast Saccharomyces cerevisiae. *Science* **344**, 1389-1392 (2014). https://doi.org:10.1126/science.1252634

13 Hanzen, S. *et al.* Lifespan Control by Redox-Dependent Recruitment of Chaperones to Misfolded Proteins. *Cell* **166**, 140-151 (2016). https://doi.org:10.1016/j.cell.2016.05.006

14 Dünkler, A. *et al.* Type V myosin focuses the polarisome and shapes the tip of yeast cells. *Journal of Cell Biology* **220**, e202006193 (2021). https://doi.org:10.1083/jcb.202006193

15 Moreno, D. F. *et al.* Competition in the chaperone-client network subordinates cell-cycle entry to growth and stress. *Life Science Alliance* **2**, e201800277 (2019). https://doi.org:10.26508/lsa.201800277
