# Supplementary material for: Artificial Hsp104-mediated systems for re-localizing protein aggregates
Source: Nat Commun. 2023 May 9;14:2663. doi: 10.1038/s41467-023-37706-3 (PMC10169802; doi:10.1038/s41467-023-37706-3)
Supplement: Supplementary file 2 — Description of additional supplementary files [file 41467_2023_37706_MOESM2_ESM.pdf]

Description of Supporting information

Supplementary Movie 1: Movie of a mHtt103QP aggregate (green, GFP labeled) going into the yeast daughter cell by using ATS1. Without ATS1, protein aggregates are usually retained in the mother cell.

Supplementary Data 1: Screening for 'inclusion generators' other than Hsp104. Raw data of screen results. GFP binding protein (GBP) was fused to Pea2 and crossed with the GFP library using SGA technology. Cells were screened for a phenotype with a strong artificial inclusion of a chimera consisting of the GFP-labelled gene of interest and Pea2. Screen data output: inclusion-to-cytoplasm-ratio of GFP signal and fraction of cells with GFP-labeled inclusions. Additionally, mHtt103QP-mCherry (GPD promoter -driven expression from a plasmid) transport to daughters was assessed.

Supplementary Data 2: Oligomerization comparison. The IGs were enriched for proteins that form homo oligomers ($p < 0.0001$, chi-square test). According to the Uniprot database, a subunit structure is known for 31% of the IGs. When focusing on homo oligomerization, 78.3% of IGs are known to be homo oligomers (3 or more subunits), whereas only 21.7% are known to be mono- or dimers. According to a recent estimation, the yeast proteome contains 28.7% homo oligomers (Danielli et al.), yielding a 2.7 fold enrichment of homo oligomers in the list of IGs. Hetero-oligomerization is probably also a driving force for the IGs, but no comparative study is known to us.
