# Supplementary material for: Artificial Hsp104-mediated systems for re-localizing protein aggregates
Source: Nat Commun. 2023 May 9;14:2663. doi: 10.1038/s41467-023-37706-3 (PMC10169802; doi:10.1038/s41467-023-37706-3)
Supplement: Supplementary file 6 — Reporting Summary [file 41467_2023_37706_MOESM6_ESM.pdf]

nature portfolio

Corresponding author(s): Thomas Nyström

Last updated by author(s): Mar 13, 2023

Reporting Summary

Nature Portfolio wishes to improve the reproducibility of the work that we publish. This form provides structure for consistency and transparency in reporting. For further information on Nature Portfolio policies, see our Editorial Policies and the Editorial Policy Checklist.

Statistics

For all statistical analyses, confirm that the following items are present in the figure legend, table legend, main text, or Methods section.

n/a	Confirmed	
☐	☒	The exact sample size (n) for each experimental group/condition, given as a discrete number and unit of measurement
☐	☒	A statement on whether measurements were taken from distinct samples or whether the same sample was measured repeatedly
☐	☒	The statistical test(s) used AND whether they are one- or two-sided<br>*Only common tests should be described solely by name; describe more complex techniques in the Methods section.*
☒	☐	A description of all covariates tested
☒	☐	A description of any assumptions or corrections, such as tests of normality and adjustment for multiple comparisons
☐	☒	A full description of the statistical parameters including central tendency (e.g. means) or other basic estimates (e.g. regression coefficient) AND variation (e.g. standard deviation) or associated estimates of uncertainty (e.g. confidence intervals)
☐	☒	For null hypothesis testing, the test statistic (e.g. F, t, r) with confidence intervals, effect sizes, degrees of freedom and P value noted<br>*Give P values as exact values whenever suitable.*
☒	☐	For Bayesian analysis, information on the choice of priors and Markov chain Monte Carlo settings
☒	☐	For hierarchical and complex designs, identification of the appropriate level for tests and full reporting of outcomes
☒	☐	Estimates of effect sizes (e.g. Cohen's d, Pearson's r), indicating how they were calculated

Our web collection on statistics for biologists contains articles on many of the points above.

Software and code

Policy information about availability of computer code

Data collection	Fluorescence microscopy image acquisition was performed with Zeiss Zen blue v2.2 or v3.1 software.<br>High content microscopy image acquisition was performed with Molecular Devices MetaXpress v6.2 software.<br>Spinnind disk microscopy image acquisition was performed with Andor Fusion 2.2.0.49 software<br>Screen data visiualization was done with STRING database (string-db.org).<br>Growth curve data acquisition was performed with Biotek Gen5 v1.7 software.<br>Flow cytometry data was collected with FACSDiva 6.1.3 software (BD)
Data analysis	Biotek Gen5 v1.7<br>Cytoscape v3.9.0<br>FIJI ImageJ 2.1.0/1.53c<br>Graphpad Prism 9<br>Microsoft excel 2016<br>Molecular Devices MetaXpress v6.2<br>DAVID Functional Annotation Tool (david.ncifcrf.gov)<br>FACSDiva 8.0.1 (BD)

For manuscripts utilizing custom algorithms or software that are central to the research but not yet described in published literature, software must be made available to editors and reviewers. We strongly encourage code deposition in a community repository (e.g. GitHub). See the Nature Portfolio guidelines for submitting code & software for further information.

Data

Policy information about availability of data

All manuscripts must include a data availability statement. This statement should provide the following information, where applicable:

- Accession codes, unique identifiers, or web links for publicly available datasets
- A description of any restrictions on data availability
- For clinical datasets or third party data, please ensure that the statement adheres to our policy

All data needed to evaluate the conclusions in the paper are present in the paper and/or the Supplementary Materials. Additional data related to this paper may be requested from the authors.

Human research participants

Policy information about studies involving human research participants and Sex and Gender in Research.

Reporting on sex and gender	Not applicable.
Population characteristics	Not applicable.
Recruitment	Not applicable.
Ethics oversight	Not applicable.

Note that full information on the approval of the study protocol must also be provided in the manuscript.

Field-specific reporting

Please select the one below that is the best fit for your research. If you are not sure, read the appropriate sections before making your selection.

☒ Life sciences ☐ Behavioural & social sciences ☐ Ecological, evolutionary & environmental sciences

For a reference copy of the document with all sections, see nature.com/documents/nr-reporting-summary-flat.pdf

Life sciences study design

All studies must disclose on these points even when the disclosure is negative.

Sample size	A minimum of 200 cells were examined for each sample for fluorescent microscopy analyses. For lifespan analysis, at least 50 cells were analyzed per replicate. No sample size calculations were performed. The here used sample size numbers provide enough single cell measurements to robustly detect major phenotypes.
Data exclusions	There were no data exclusions.
Replication	The experiments were performed in three independent experiments unless specified. Lifespan experiments were performed in two independent experiments. All attempts to repeat the experiment were successful.
Randomization	Lifespan experiments were randomized. The other experiments were not randomized, since there was no allocation into subgroups.
Blinding	Lifespan experiments were blinded. The other experiments were not blinded as there was no allocation into subgroups.

Reporting for specific materials, systems and methods

We require information from authors about some types of materials, experimental systems and methods used in many studies. Here, indicate whether each material, system or method listed is relevant to your study. If you are not sure if a list item applies to your research, read the appropriate section before selecting a response.

Materials & experimental systems

n/a	Involved in the study
☒	☐ Antibodies
☐	☒ Eukaryotic cell lines
☒	☐ Palaeontology and archaeology
☒	☐ Animals and other organisms
☒	☐ Clinical data
☒	☐ Dual use research of concern

Methods

n/a	Involved in the study
☒	☐ ChIP-seq
☐	☒ Flow cytometry
☒	☐ MRI-based neuroimaging

Eukaryotic cell lines

Policy information about cell lines and Sex and Gender in Research

Cell line source(s)	HEK293 cells were obtained from the American Tissue Culture Collection (ATCC).
Authentication	No further authentication was conducted.
Mycoplasma contamination	All cell lines tested negative for Mycoplasma contamination using the Eurofins Mycoplasma testing service.
Commonly misidentified lines (See ICLAC register)	No commonly misidentified lines were used.

Flow Cytometry

Plots

Confirm that:

☒ The axis labels state the marker and fluorochrome used (e.g. CD4-FITC).

☒ The axis scales are clearly visible. Include numbers along axes only for bottom left plot of group (a 'group' is an analysis of identical markers).

☒ All plots are contour plots with outliers or pseudocolor plots.

☒ A numerical value for number of cells or percentage (with statistics) is provided.

Methodology

Sample preparation	Log-phase cells, grown in SD-Ura with 2% raffinose, were labeled with 5 µg/ml sulfo-cyanine5-NHS ester (Lumiprobe) in PBS for 10 min at RT. Cells were washed twice with PBS. Subsequently, the cells were grown for 1 h in SD-Ura with 2% raffinose and then induced with 1% galactose for 3.5 h. After a wash with PBS and cell death staining with 0.4 µg/ml DAPI (ThermoFisher Scientific), the cell death rate was determined with flow cytometry.
Instrument	Becton Dickinson (BD) LSR Fortressa
Software	FACSDiva 8.0.1
Cell population abundance	Three to four independent experiments were performed with 30,000 Cyanine5-positive cells being evaluated.
Gating strategy	Yeast cells were defined based on light scatter using FSC-area vs. SSC-area on a logarithmic scale. Doublet discrimination was performed by plotting the width against the area for forward scatter and side scatter. Cyanine5-positive cells were detected based on the signal in the APC channel (640nm excitation; 670/14 nm bandpass). Dead cells were labelled with DAPI and then detected in the HOECHST channel (355nm excitation; 450/50 nm Bandpass).

☒ Tick this box to confirm that a figure exemplifying the gating strategy is provided in the Supplementary Information.
